# Outcome of physiotherapy after surgery for cervical disc disease: a prospective randomised multi-centre trial

**DOI:** 10.1186/1471-2474-15-34

**Published:** 2014-02-06

**Authors:** Anneli Peolsson, Birgitta Öberg, Johanna Wibault, Åsa Dedering, Peter Zsigmond, Lars Bernfort, Ann-Sofi Kammerlind, Liselott CG Persson, Håkan Löfgren

**Affiliations:** 1 Department of Medical and Health Sciences, Physiotherapy, Faculty of Health Sciences, Linköping University, SE-58183 Linköping, Sweden; 2 Department of Physical Therapy, Karolinska University Hospital, Stockholm, Sweden; 3 Department of Neurobiology, Care Sciences and Society, Division of Physiotherapy, Karolinska Institutet, Stockholm, Sweden; 4 Department of Neurosurgery, Linköping University Hospital, Linköping, Sweden; 5 Department of Medical and Health Sciences, Health Care Analysis, Linköping University, Linköping, Sweden; 6 Futurum the Academy for Healthcare, County Council Jönköping, Jönköping, Sweden; 7 Division of Physiotherapy, Department of Health Sciences, Lunds University, Lund, Sweden; 8 Neuroorthopedic Centre, Ryhov Hospital, Jönköping, Sweden

**Keywords:** Intervertebral disc, Spine, Neck, Rehabilitation, Physical therapy

## Abstract

**Background:**

Many patients with cervical disc disease require leave from work, due to long-lasting, complex symptoms, including chronic pain and reduced levels of physical and psychological function. Surgery on a few segmental levels might be expected to resolve disc-specific pain and reduce neurological deficits, but not the non-specific neck pain and the frequent illness. No study has investigated whether post-surgery physiotherapy might improve the outcome of surgery. The main purpose of this study was to evaluate whether a well-structured rehabilitation programme might add benefit to the customary post-surgical treatment for cervical disc disease, with respect to function, disability, work capability, and cost effectiveness.

**Methods/Design:**

This study was designed as a prospective, randomised, controlled, multi-centre study. An independent, blinded investigator will compare two alternatives of rehabilitation. We will include 200 patients of working age, with cervical disc disease confirmed by clinical findings and symptoms of cervical nerve root compression. After providing informed consent, study participants will be randomised to one of two alternative physiotherapy regimes; (A) customary treatment (information and advice on a specialist clinic); or (B) customary treatment plus active physiotherapy. Physiotherapy will follow a standardised, structured programme of neck-specific exercises combined with a behavioural approach. All patients will be evaluated both clinically and subjectively (with questionnaires) before surgery and at 6 weeks, 3 months, 6 months, 12 months, and 24 months after surgery. The main outcome variable will be neck-specific disability. Cost-effectiveness will also be calculated.

**Discussion:**

We anticipate that the results of this study will provide evidence to support physiotherapeutic rehabilitation applied after surgery for cervical radiculopathy due to cervical disc disease.

**Trial registration:**

ClinicalTrials.gov identifier: NCT01547611

## Background

Patients with cervical disc disease (herniation and/or spondylotic changes) often present complex symptomatology. The symptoms include disc-specific and non-specific neck pain, distinct, intense arm pain, sensory loss, motor loss, and reflex abnormalities. Furthermore, the symptoms are often followed by physical and psychological disability, illness, long periods of sick-leave, and difficulty returning to work [1-6].

Surgeries aimed to decompress the spinal nerve root and/or medulla have been established worldwide for managing radiculopathy (annual incidence is about 0.8% [7]) due to cervical disc disease [2,8,9]. Several studies have reported that surgeries reduced pain intensity and neurological deficits, and the overall outcome was good in approximately 80% of cases [9]. However, when broader, more functional measurements were evaluated, the results were less favourable [2,4,6,10,11].

Few studies are available from prospective, randomised studies on patients with cervical disc disease that received decompressive surgery with long-term follow-ups and functional measurements [12,13]. Peolsson et al. [13] reported that neck-specific function was not improved in an average six-year follow-up. Those patients also reported poor health-related quality of life (EQ-5D 0.61) [13] after surgery, worse than that reported by patients with low-back pain or patients with asthma. Moreover, over one third of patients displayed physical deficits, including decreased range of neck motion, reduced neck and hand muscle strength [14], and reduced neck muscle endurance [3]. About two-thirds of patients reported high intensity neck pain, neck-specific disability, psychological distress, and poor general health [14]. Also, after spinal surgery, health status tended to be worse in women than in men [3,14]. At a 3-year follow-up after surgery, 83% of patients reported neck disorders, and 63% of those reported daily pain [14]. At one year after surgery, 56% of patients were on full-time sick-leave, and 12% were on part-time sick-leave [15]. In another prospective randomised study, at follow-ups conducted 10–13 years after surgery, more than one third remained on sick-leave related to the neck [12].

Surgery on one or a few segmental levels is expected to cure cervical disc-specific pain and reduce neurological deficits, but not non-specific neck pain or related illnesses. Patients with cervical disc disease are excluded from most physiotherapy studies, due to their “specific” diagnoses, despite their presentation of non-specific neck pain. Moreover, these patients are often considered to have chronic pain. These circumstances have resulted in the need for a structured physiotherapy programme designed to improve physical and psychological function, facilitate performance of daily activities, and teach pain management.

The effect of physiotherapy treatment is not well documented for patients with cervical disc disease and symptoms of cervical radiculopathy [5,11,16-19]. Moreover, there is a paucity of well-defined, structured physiotherapy programmes. Persson et al. [5] compared surgery with pragmatic physiotherapy and found no long-term differences between treatment strategies. Peolsson [11] and Engquist [19] investigated the benefit that surgery might add to a structured physiotherapy programme. Apart from lower neck pain at the 2-year follow-up [19], they found no differences between groups in pain or neck specific function, whether self-rated or observer-rated in a neck-related function test [11,19]. Nevertheless, there is a lack of randomised controlled studies that evaluated a physiotherapeutic rehabilitation programme in patients with cervical radiculopathy. No study has investigated whether post-surgery physiotherapy might improve the outcome of surgery.

Clinical experience has shown that patients with cervical disc disease commonly experience dizziness and difficulty balancing, but scientifically, little is known about this phenomenon. In treating individuals that experienced pain and disability after a whiplash trauma to the neck and other painful conditions, important factors for recovery include the patient’s self-efficacy and use of coping strategies [20-22]. Self-efficacy is defined as “a person’s belief in his or her ability to succeed in a particular situation” [23]. Coping is defined as “the constantly changing cognitive and behavioural adjustments made in an effort to manage specific external or internal demands” [24]. No report on patients with cervical disc disease has investigated self-efficacy in daily activities or coping with a neck-related disability. Furthermore, no report on patients with cervical disc disease has assessed work capability or satisfaction with care.

The main purpose of this study was to evaluate whether a well-structured rehabilitation programme might add benefit to customary treatment after surgery for cervical disc disease, with respect to function, disability, work capability, and cost effectiveness.

## Methods/Design

### Study population

After informed consent patients (n=200) will be included from four Neurosurgical clinics in the south of Sweden and the rehabilitation will be performed in primary care units in several counties.

#### Eligability criteria

Inclusion criteria for surgery: Cervical disc disease, verified with magnetic resonance imaging (MRI) and compatible with clinical findings (examined by a neurosurgeon) that show cervical nerve root compression; Radiculopathy; At least 2 months of persisting nerve root pain.

Inclusion criteria for the study: Surgery for cervical disc disease (an anterior surgical decompression and fusion or posterior surgery, foraminotomy/ laminectomy) in one to three segmental levels) Age 18–70 years.

Exclusion criteria: Myelopathy; Previous fracture or luxation of the cervical column; Malignancy or spinal tumour; Spinal infection; Previous surgery in the cervical column; Systematic disease or a trauma that contraindicates the performance of either the treatment programme or the measurements; Diagnosis of a severe psychiatric disorder, such as schizophrenia or psychosis; Known drug abuse; Lack of familiarity with the Swedish language.

### Design

This is a prospective, randomised, controlled multi-centre study with an independent, blinded investigator to compare two alternative rehabilitation programmes. After inclusion, the patients will be randomised to receive either (A) customary treatment, including information and advice at the Neurosurgery clinic (n = 100); or (B) customary treatment plus a standardised, structured, behavioural medicine programme. The programme will start 6 weeks after surgery and continue for 20 weeks (n = 100). Randomisation (a random computer list created by a university statistician) will be performed by the central project leader.

### Interventions

Both treatments will be performed by primary care physiotherapists with specific competence and interest in patients with neck pain. To measure treatment compliance in both groups, a diary will be maintained that records all the exercises performed, educational segments undertaken, and the patient’s progress in each segment.

**Group A: customary treatment.** The staff at the Neurosurgical clinic will give the patient standard pre- and postoperative information. The physiotherapist at the Neurosurgical clinic will inform the patient about movements to avoid during the first weeks after surgery and the importance of good posture and ergonomic thinking in daily life. The patient will also be instructed on the proper performance of shoulder exercises for keeping range of motion. About 6 weeks after the surgery, patients will make routine post-surgery visits for examinations by the surgeon and the physiotherapist. The physiotherapist will instruct the patient in the proper performance of exercises that involve active neck range of motion.

**Figure 1:**
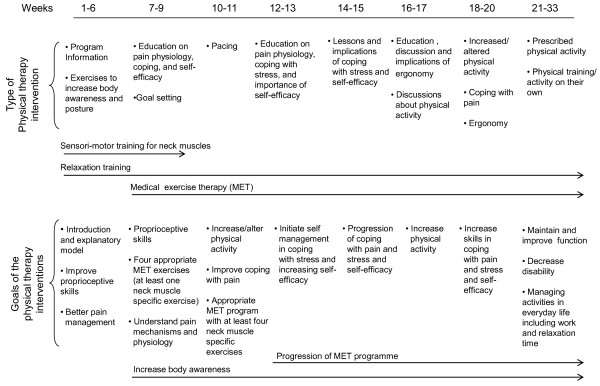
**Flow-chart of the standardised, structured physiotherapy programme.** Note: Therapy week 1 begins at 6 weeks after surgery.

**Group B: customary treatment (above) plus a standardised, structured, behavioural medicine programme** (Figure 1). The behavioural medicine programme includes a functional behavioural analysis of the problem, medical exercise therapy, strategies to increase self-efficacy in activities, and problem-solving strategies for coping with disability.

The programme was designed based on recent evidence from treatments of patients with other kinds of neck disorders or with long-standing musculoskeletal pain [25-28]. Most patients that undergo surgery for cervical disc disease have had severe pain for a few years (mean duration: 3 years), and they have commonly developed widespread pain before the neck surgery. The goal of the treatment is to improve function, improve performance of basic activities, increase self-efficacy, and teach constructive strategies for coping with pain and disability.

The medical exercise therapy focuses on sensorimotor training, neck stabilisation, neck muscle endurance, and strengthening the muscles that stabilise the scapula. Additionally, throughout the programme, the patient will work on postural correction and ergonomics. Based on a well-defined frame of exercises that provide a standardised, structured progression, the experienced physiotherapist will adjust the programme by selecting exercises and dosages appropriate for each patient (after a clinical examination and functional behavioural analysis). The programme starts 6 weeks after surgery. The patient will begin with sensorimotor training once a week in the physiotherapy clinic and daily exercises at home that include sensorimotor training and relaxation techniques. Twelve weeks after surgery, the patient starts exercises twice a week for stabilisation, muscle endurance, and strength [29]; these will be performed for 14 weeks (weeks 12–25 after surgery). Thereafter, patients will be encouraged to continue the exercises and to increase their overall activity level, according to a physical activity routine provided by the physiotherapist.

During the 20 weeks of treatment, once a week, the patient and the physiotherapist will discuss topics concerning pain physiology, the consequences of stress, the importance of gradually increasing exercise intensity, different breathing techniques, coping strategies, pacing, and ergonomics. The patient will also learn how to cope with the physical and psychological consequences of pain and disability. This training will be enhanced by continuing the relaxation training, performing exercises that increase body awareness, setting goals for activities, and increasing self-efficacy in daily activities.

Each physiotherapist included in the study will be introduced and educated to the programme by the project leader. The treatment approach will be well-documented in a treatment manual, which will be given to each physiotherapist. Written treatment material will also be given to patients.

### Clinical measurements performed by independent, blinded evaluators

The measurements will be performed before surgery and at 3, 6, 12, and 24 months after surgery. The neck muscle endurance test will not be performed at 3 months after surgery. The selected measurements have previously shown good reliability, and known reference values are available for healthy individuals.

#### Clinical measurements

Neurological exam to test cervical nerve roots (sensibility, motor function, reflexes); Range of motion of the neck in all three planes, measured with the plastic helmet, cervical range of motion device (CROM) [30]; Head repositioning accuracy, measured with the CROM [31]; Anterior and posterior neck muscle endurance, measured in seconds [32]; Hand strength, measured with the Jamar hand-dynamometer [33]; Static and dynamic clinical balance tests; static balance measured in the sharpened Romberg position, with the non-dominant foot in front of the dominant foot and eyes closed [34]; dynamic balance measured as the patient walks in a figure-eight pattern [35].

Patient self-rated ability to perform important activities, measured with the Patient-Specific Functional Scale [36]; the scale will be filled out by the test-leader in dialogue with the patient.

### Questionnaires

The patients will complete questionnaires that provide background data, disease-specific data, and generic data. These questionnaires will be completed before surgery and at 6, 12, and 24 months after surgery. Additionally, shortened questionnaires will be completed at 6 weeks and 3 months after surgery. The selected questionnaire instruments have previously shown good reliability and validity.

#### Background questionnaires

*Personal characteristics*: Gender, age, social situation, smoking habits, presence of back pain, current pain medications; *Pain history*: Pain related to the neck complaint, including initiation, duration, and localisation; previous medical problems (including differential-diagnoses for cervical disc disease); earlier treatments for the neck complaint or its effects; *Environment*: Work situation, including the type of work, the workload to the neck, and work satisfaction; physical activity/exercise habits.

#### Main outcome

Neck specific disability, measured with the Neck Disability Index [37].

#### Secondary outcomes

Neck pain intensity, arm pain, headache, and dizziness, all measured with a Visual Analogue Scale (VAS) (0–100 mm) [34,38]; Pain distribution, measured on a drawing, where the patient indicates symptom locations [39]; Self-efficacy in daily activities, measured with the Self-efficacy Scale [40]; Symptom satisfaction, related to neck problems (how would the patient feel about experiencing the current neck symptoms for the rest of his/her life), rated on a seven-grade scale [41]; Psychological and psycho-somatic distress, measured with the Distress and Risk Assessment Method (DRAM) [42]; Coping strategies, measured with the Coping Strategies Questionnaire (CSQ) [43]; Work capability, measured with the Work Ability Index (WAI) [44]; Sick-leave, measured as the number of days off work; Health related quality of life, assessed with the EuroQol five dimensions self-report (EQ-5D) and Current health status, measured with the EuroQol vertical VAS (0–100 mm) [45]; Impact on social relationships, measured with the West Haven Multidimensional Pain Inventory, Swedish version (MPI-S), the MPI-S for impact on significant others, and open questions [46].

### After surgery

After surgery, *additional* information will be collected to determine patient fulfilment and their satisfaction with the treatment:

Global outcome of surgery, measured with a modified Odom scale, scored on a six-point scale; The importance of the improvements provided by the surgery, measured with a VAS; Satisfaction with information and care given after the surgical intervention, measured with the Patient Enablement Instrument and open questions [47]; Questions regarding the re-surgery, when applicable; Vocational situation, measured with a questionnaire and interviews. For example, the subjects will be asked about the present work situation, hours worked, and type of contract. They will also be asked about their intention to continue working or return to work. They will also be asked about whether they expect to remain in their present occupation and work place.

### Cost-effectiveness

Calculating the cost-effectiveness of an intervention requires data on both the effects and the costs. Costs are divided into direct costs and indirect costs. Direct costs are directly associated with the intervention, and mainly consist of health care costs. In this study, data on health care costs, mainly the quantity and type of health care visits, will be collected from a health care registry and by asking the patients. Indirect costs are incurred due to the negative effects of an intervention. These mainly consist of production loss, because patients are unable to perform their work due to ill health. Costs associated with the inability to work are calculated according to economic theory; i.e., gross income, plus taxes, for the time of absence from work. Data on sick-leave and income will be collected from the Social Insurance Office. The effects of an intervention are measured in terms of the change in quality of life. In health economic evaluations, costs are most often related to effects in terms of quality-adjusted life years (QALYs).

The cost-effectiveness of an intervention (int) is calculated by comparing it to an alternative (alt), in this case the “customary treatment”, as follows: (Costs_int_-Costs_alt_)/(Effects_int_-Effects_alt_).

### Ethical considerations

This study will be conducted in accordance with the declaration of Helsinki and with Swedish laws. The Ethics Committee at the Faculty of Health Sciences at Linköping University, Linköping in Sweden (Dnr-M126-08 and M126-08 T99-09) has approved the study. Written informed consent will be obtained from all patients included in the study. Patients will be informed that they are free to leave the study, without explanation and without any negative consequences on future treatment. There are no known risks associated with patient participation in the study, except possible temporary muscle-aches after exercise. All physiotherapists involved in the study will be registered at the National Board of Health and Welfare in Sweden. All personal patient details will be rendered anonymous before data-entry. There are no commercial interests tied to this study.

### Statistical analysis and power calculation

Earlier studies have suggested that 60 patients will be required for each treatment arm to provide sufficient statistical power. However, no previous study has compared two physiotherapy approaches applied after surgery in this patient category. Earlier studies have shown that, after surgery, approximately one third of patients experienced great improvement or complete relief from neck complaints. Based on those results, we estimated that 100 patients will be required for each treatment arm to provide sufficient statistical power for the study (200 patients, total). Data will be analysed according to an intention-to-treat approach. An alternative analysis will be performed to take treatment compliance into consideration. Analyses will be performed with parametric or non-parametric statistics, depending on the type of data. The type of surgery will be included as a co-variant in the statistical analysis.

### Gender perspectives

Both males and females will be included in the study. When applicable in the study, the impact of gender will be specifically analysed and presented in the results. Currently, more males than females undergo surgery annually for cervical disc disease. However, compared to males, females have exhibited worse results and reported more problems that persisted after surgery [14]. Thus, female gender appeared to be a negative predictor for a good surgical outcome [3]. We lack knowledge to explain this tendency and to formulate ways improve it. It is therefore of great importance to expand our knowledge on gender differences to be able to improve rehabilitation after surgery.

### Time frame

The estimated time period for patient inclusion in the study is 4 years. Follow-up will continue for another 2 years.

## Discussion

Among patients that have undergone surgery for cervical disc disease, as much as two thirds experienced residual physical and psychological disabilities and about half remained out of the labour market for long periods after surgery. A majority of patients experienced widespread pain. Patients that have undergone surgery for cervical disc disease had a mean age of about 46 years; i.e., they were in the middle of their working career.

Most studies in the field have not focused on function, participation, or rehabilitation. Instead, they focused on surgical techniques. Currently, the outcome of post-surgery physiotherapy is not known for patients treated with surgery for cervical disc disease. If it were found that surgery combined with an active, structured physiotherapy programme could add benefit over surgery combined with only physiotherapeutic advice, then an appropriate programme would facilitate the direction of patient rehabilitation after surgery.

The active rehabilitation model will consist of neck-specific exercises and a pain- and stress-management model inspired by a cognitive behavioural approach. This is consistent with existing evidence for treating long-standing neck problems that originate from causes *other* than cervical disc degenerative disease [25]. To date, no neck exercise therapies or cognitive behavioural approaches have been evaluated scientifically for patients after surgery for cervical disc disease.

## Conclusions

The present study design is unique and innovative. The results of this study may facilitate clinical decision making, improve health care, reduce physical, mental, and social costs for the patients, and reduce the cost for society. Finally, the results are expected to provide evidence in favour of physiotherapeutic rehabilitation after surgery for cervical radiculopathy due to cervical disc disease.

## Competing interests

The authors declare that they have no competing interests.

## Authors’ contributions

AP initiated the study and was responsible for the overall design of the study. BÖ, LB, ASK, PZ, and HL are experts in their respective fields and critically discussed the study design with AP. As project leader, AP had the main responsibility of applying for funding, but HL and JW also applied for funding. AP, JW, ÅD, LP, and HL were responsible for data collection. Data analysis will be performed mostly by AP and JW, with support from a statistician. AP wrote the manuscript. All authors read, revised, and approved the final manuscript.

## Pre-publication history

The pre-publication history for this paper can be accessed here:

http://www.biomedcentral.com/1471-2474/15/34/prepub
